# The Burden of Deep Vein Thrombosis and Risk Factors in Pregnancy and Postpartum—Mirroring Our Region’s Particularities

**DOI:** 10.3390/jcm13164705

**Published:** 2024-08-11

**Authors:** Catalina Filip, Sofia Alexandra Socolov, Daniela Roxana Matasariu, Alexandra Ursache, Karina Delia Pisla, Tudor Catalin Gisca, Elena Mihalceanu, Vasile Lucian Boiculese, Demetra Socolov

**Affiliations:** 1 Department of Vascular Surgery, University of Medicine and Pharmacy ‘Gr. T. Popa’, 700115 Iasi, Romania; filipcatalina20@gmail.com; 2 CHU “Gabriel Montpied”, 63000 Clermont-Ferrand, France; 3 Department of Neurology, Emergency Hospital ‘Professor Doctor Nicolae Oblu’, 700309 Iasi, Romania; sofia.socolov@yahoo.com; 4 Department of Obstetrics and Gynecology, University of Medicine and Pharmacy ‘Gr. T. Popa’, 700115 Iasi, Romania; gisca_tudor-catalin@d.umfiasi.ro (T.C.G.); elena.mihalceanu@umfiasi.ro (E.M.); demetra.socolov@umfiasi.ro (D.S.); 5 Department of Obstetrics and Gynecology, Cuza Voda Hospital, 700038 Iasi, Romania; 6 Faculty of General Medicine, University of Medicine and Pharmacy ‘Gr. T. Popa’, 700115 Iasi, Romania; karina-delia.ed.pisla@students.umfiasi.ro; 7 Biostatistics, Department of Preventive Medicine and Interdisciplinarity, “Grigore T. Popa” University of Medicine and Pharmacy, 700115 Iasi, Romania; lboiculese@gmail.com

**Keywords:** deep vein thrombosis, pregnancy, pulmonary embolism, obesity, thrombophilia, diabetes, hypertension, antiphospholipid syndrome, systemic lupus erythematosus, venous insufficiency

## Abstract

(1) **Background:** The three factors within the Virchow triad play the leading role in the development of deep vein thrombosis (DVT) during pregnancy. (2) **Methods:** This research approaches the various risk factors associated with DVT and its most representative complications, pulmonary thromboembolism and cerebral venous thrombosis, in pregnant and postpartum women across a 15-year period (2007–2021). (3) **Results:** A total of 201 out of 287 patients with DVT had associated risk factors, while 86 did not present with any. Out of the 201 patients with risk factors, 47 developed pulmonary thromboembolism, while 12 experienced cerebral thrombosis. The statistical analysis of risk factors involved in DVT revealed high significance for obesity (OR 3.676; CI 2.484–5.439), gestational diabetes (OR 3.394; CI 2.101–5.483), hypertension (OR 2.325; CI 1.591–3.397), preeclampsia (OR 4.753; CI 2.342–9.645), thrombophilia (OR 12.138; CI 8.973–16.417), and varicose veins (OR 9.678; CI 7.321–12.793); for pulmonary thromboembolism, there was high significance for obesity (OR 7.867; CI 4.297–14.401), hypertension (OR 2.605; CI 1.246–5.446), preeclampsia (OR 7.483; CI 2.346–23.872), thrombophilia (OR 11.035; CI 5.910–20.602), and varicose veins (OR 6.837; CI 3.665–12.757); and for cerebral thromboembolism (CTE), the risk factors identified were obesity (OR 6.755; CI 1.954–23.347), hypertension (OR 1.167; CI 0.155–8.770), preeclampsia (OR 9.655; CI 1.283–72.672), and thrombophilia (OR 33.275; CI 12.884–85.939). (4) **Conclusions:** Obesity was the only significant factor found to influence DVT, pulmonary embolism and CTE risks, and hereditary thrombophilia was the main factor influencing the risk for pulmonary thromboembolism and CTE. Systemic lupus erythematosus and gestational diabetes revealed conflicting results that require further investigation.

## 1. Introduction

Structural elements aligning with the Virchow triad contribute to the pathogenesis of deep vein thrombosis (DVT) during pregnancy. In certain cases, an imbalance can arise between the functionality of naturally existing antithrombotic mechanisms and pro-coagulant ones. In actual medical practice, there are inherited and achieved factors that can promote coagulation due to genetically determined or acquired conditions [1]. Coexisting pathologies can considerably increase the risk of DVT [2,3].

In contrast with women who are not pregnant, those who are pregnant or up to 6 months in the postpartum period face a heightened risk of venous thromboembolism, resulting in a global incidence of around 0.1%. Additionally, women with a history of venous thromboembolism encounter elevated risks (ranging from 2% to 10%) of experiencing recurrent episodes during pregnancy and postpartum compared to women without such a history [4]. Thrombophilia, a common hereditary condition, is among the most frequent causative factors documented in the literature [2,5]. Pregnant and postpartum women are at a heightened risk of thrombotic events, even more so in cases that undergo emergency cesarean section due to various reasons [6]. 

The left-sided predominance of thrombosis during pregnancy is associated with hormonal changes that determine vasodilatation starting from the first trimester, which, in late pregnancy, are associated with uterine compression that determines vascular stasis, especially in the lower limbs [7]. The most frequent sites of thrombosis during pregnancy or up to 6 months postpartum are the left iliofemoral vein, with an incidence ranging from 70% to 90% of cases, and the iliac vein. In almost 80% of cases, there is a unilateral inferior limb involvement [8,9]

Other often-encountered conditions strongly correlated with Virchow’s triad and that increase the risk of thrombosis are obesity and hypertension disorders (pregnancy-induced hypertension, preeclampsia, eclampsia, and HELLP syndrome) [10,11,12]. Another important participant that enhances thrombotic risk in pregnancy and postpartum is diabetes, whether preexisting or gestational. The association of multiple factors such as diabetes, together with obesity and/or maternal age, further amplifies this risk [13]. Thrombotic events are often encountered in cases involving antiphospholipid and systemic lupus anticoagulant antibodies [14]. Additional risk factors can also include advanced maternal age, multiparity, multiple pregnancies, blood group type I, and smoking [15,16].

Abounding data in the literature underline the different impacts between ethnic and racial groups concerning the incidence of DVT. These differences have not yet been completely explained due to the complex interactions between environmental and genetic factors, with lower rates of venous thromboembolism being found in Asiatic populations and higher rates noted in Hispanic and European populations. The African American population remains the group most predisposed to such complications [17,18].

The clinical manifestations of DVT in pregnant or postpartum women encompass lower extremity discomfort, leg edema, and abdominal or lower back pain [19]. Timely diagnosis and fast initiation of treatment are imperative to prevent complications that can result in pulmonary or cerebral embolism and post-thrombotic syndrome [20,21].

Despite its rarity, DVT, during or after pregnancy, significantly impacts maternal mortality and morbidity due to the pro-coagulant nature of pregnancy. DVT and its complications affect the quality of life of these women, not mentioning the significant negative impacts following a thrombotic episode. Our study represents the most extensive research conducted in our country to date, marking it as the second investigation intending to address this particular subject [22]. This research aims to assess the prevalence of DVT in our region, as well as the complications the disease poses during pregnancy and postpartum women in relation to various risk factors. We intend to determine whether we can individualize the particularities of our DVT population with the aim of adapting and adjusting our prophylaxis measures to reduce maternal morbidity and also identify the major associated pathologies in our region for most thrombotic events. 

## 2. Materials and Methods

To initiate our investigation, three distinct cohorts of patients, all comprising females within the fertile age range, were recruited from three separate medical clinics situated in Iaşi. The initial cohort consisted of 200 patients diagnosed with deep vein thrombosis out of a total of 91,909 patients who underwent childbirth at the Obstetrics and Gynecology Clinical Hospital “Cuza Voda” in Iaşi between 2007 and 2021. The second group was composed of 18 pregnant women or those in a recent postpartum period diagnosed with deep vein thrombosis in the cerebral venous territory. The group was extracted from a subset of 91 patients with the same pathology sourced from the Clinical Neurology Emergency Hospital “Prof. Dr. Nicolae Oblu” in Iaşi, during the aforementioned period. The last study cohort involved 69 pregnant or postpartum women diagnosed with deep vein thrombosis out of a larger cohort of 568 patients that shared the same diagnostic profile, sourced from the “Sf. Spiridon” Emergency County Hospital in Iaşi. 

The initial phase of participant selection involved the utilization of electronic patient records from the designated hospitals. The patients that met the predefined criteria were systematically identified.

The inclusion criteria comprised pregnant women or those up to 6 months postpartum, identified with the assistance of an index cataloging all births occurring at the Obstetrics and Gynecology Clinical Hospital “Cuza Voda” in Iasi during specified annual periods. Furthermore, cases of interest were discerned utilizing keywords such as “thrombosis”, “obesity”, “diabetes”, “hypertension”, “preeclampsia”, “varicose veins”, “systemic lupus erythematosus”, and “other coagulation disorders”, adhering to the WHO ICD 9 and 10 codifications for thrombophilia. The medical records of selected cases underwent meticulous review, and supplementary information concerning clinical manifestations, diagnostic techniques, birthing methods, complications, and treatment modalities was retrieved. All of the cases from the study cohorts identified from “Prof. Dr. Nicolae Oblu” and “Sf. Spiridon” Clinical Emergency Hospitals in Iaşi were selected with the aid of ICD 9 and 10 diagnostic codes linked to deep vein thrombosis and its complications, followed by a second selection step that resulted in our target group, comprising only pregnant or postpartum women. The ICD 9 and 10 codes we used to identify and select our cases were as follows: O22.3, O22.5, O72.3, E88.2, E66.0, E11.9, E10.9, O24.41, 026.0, D68.8, D68.1, D68.2, D68.4, O13, O10.0, O14.0, and O22.0. The postpartum period elected for the inclusion of the patients was 6 months after childbirth.

All of the patients enrolled in our study agreed and signed the informed consent form approved by the Ethical Committee of the University of Medicine and Pharmacy “Gr. T. Popa”, Iaşi, no. 105/22.7.2021.

After our selection process, an extended study group encompassing 91,996 patients aged between 12 and 53 was examined, among whom 287 were diagnosed with deep vein thrombosis and associated complications. Our study cohort encompassed pregnant or recently postpartum women (following vaginal or cesarean delivery) aged between 16 and 46 years that had a confirmed diagnosis of deep vein thrombosis, with or without associated risk factors. Conversely, the exclusion criteria involved insufficient data from the observational study and suspected thrombosis lacking diagnostic certainty. 

Our primary endpoints were to detect the incidence of DVT in pregnancy and up to 6 months postpartum and to evaluate the impact of coexisting pathologies on DVT risk. As secondary endpoints, we strove to rank our identified risk factors and underline ethnic and racial particularities.

To ensure the reliability and consistency of our DVT diagnosis, we used the LEFt (left leg, edema, first-trimester presentation) score. If the LEFt score indicated improbability, d-dimer levels were measured, and compression ultrasonography was performed. For patients with a high suspicion of DVT, an initial compression ultrasonography was performed, and if the evaluation was positive, the diagnosis of DVT was confirmed, and treatment was initiated. If the suspicion was not high, serial testing was performed over the next 7–14 days [23]. The medical personnel involved in diagnosing DVT received regular training to ensure the correct application of the diagnostic criteria and guarantee that the diagnosis was consistently and reliably performed throughout the study.

### Statistical Analyses

The data were preprocessed in Microsoft Excel 2016 and then analyzed in SPSS 24 (IBM Corp. Released 2016. IBM SPSS Statistics for Windows, Version 24.0., Armonk, NY, USA: IBM Corp.). We conducted a univariate analysis after presenting the data in a descriptive manner. 

We used similar methods, namely, logistic regression and contingency tables, in the univariate and multivariate analysis. We used the regression line’s coefficients to compute the odds ratio (OR) in its raw, unadjusted form. Afterward, we evaluated the level of significance and the confidence intervals for each of the aforementioned risk factors to identify their level of implication in DVT, cerebral thrombosis, and pulmonary thromboembolism. We applied the forward selection method with entry testing based on the significance of the score and removal testing based on the probability of a likelihood ratio (the maximum partial likelihood estimates).

We aimed to elucidate the impact of coexisting pathologies on the risk of DVT and to conduct a ranking assessment of these factors within our population. Furthermore, we intend for our research to contribute to the existing body of literature by enhancing our understanding of ethnic and racial disparities in relation to the incidence of DVT. 

## 3. Results

After we analyzed 91,996 pregnant and recently postpartum women across a 14-year time frame (2007–2021), we noted some distinctive demographic characteristics. The statistical analysis performed to identify the annual distribution of the cases revealed a range of 5184 to 6870 births per year, with a median of 6158. As anticipated, these data, directly reflecting the incidence of deep venous thrombosis, suggest that higher birth rates correlate with an elevated risk of diagnosis.

**Figure 1:**
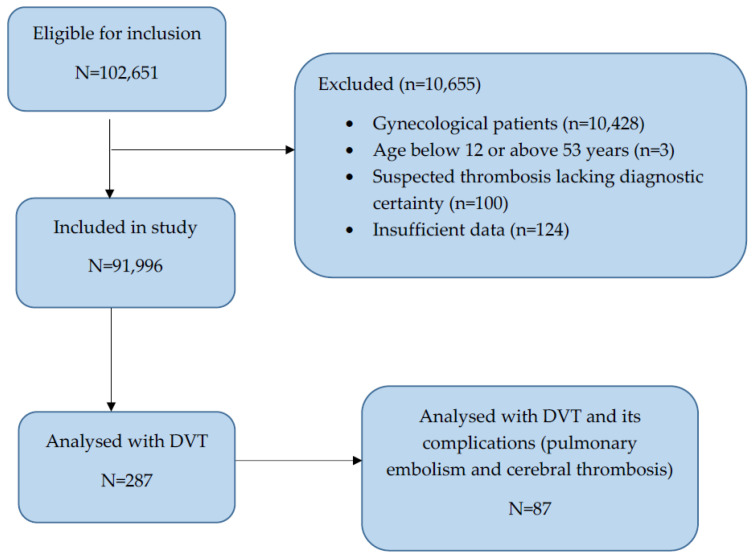
Patient recruitment flowchart.

We identified 200 cases from the Obstetrics and Gynecology Clinical Hospital “Cuza Vodă”, Iaşi; there were four pregnant patients and fourteen postpartum patients from the “Prof. Dr. Nicolae Oblu” Emergency Clinical Hospital. For the Emergency County Clinical Hospital “Sf. Spiridon”, there were 28 pregnant patients and 41 in the postpartum period. Our study cohort comprised a total of 287 patients when focusing on pregnant/postpartum individuals, resulting in a DVT prevalence of 0.31% (Figure 1).

Pregnant and recently postpartum women aged between 31 and 40 years old had the highest incidence of DVT (1.2%), with a 1.8% risk of developing the pathology (95% CI: 1.4–2.2). We observed a slight increase in the incidence of this pathology during our analyzed time frame of 0.05% (95% CI: 0.02–0.08%) per year, peaking in 2018.

The detailed general characteristics of the 91,996 patients are outlined in Table 1. When analyzing all the patients screened, we detected a total of 13,984 (15.2%) found to have concomitant risk factors, encompassing conditions such as obesity, gestational diabetes, hypertension, systemic lupus erythematosus, hereditary thrombophilia, and inferior limb varicose veins, associated with venous insufficiency. Concerning antiphospholipid syndrome and systemic lupus erythematosus, we detected a total of 84 cases (0.091%) among the 91,996 evaluated patients. Because the diagnostic criteria for thrombophilia were not widely accepted, 101 patients with this condition were identified from the available medical data during the analysis period of 2007–2011. Subsequently, after 2011, due to a more rigorously implemented coding system, 1629 cases met the diagnostic criteria. The total number of patients diagnosed with thrombophilia over the entire period was 1730, representing 1.88% of births and 13.04% of pulmonary embolism cases. A total of 2758 patients (2.99%) were diagnosed with varicose veins of the lower limbs. Out of the 287 patients with DVT, 22.64% had associated varicose veins. 

Table 2 outlines the general characteristics of the deep vein thrombosis group. The mean age of patients with deep vein thrombosis is 31.3 years. Among these, 185 (64.45%) were pregnant at the time of diagnosis, and 102 (35.54%) were in the postpartum period. The dominant site for deep vein thrombosis was the left lower limb (45.2%). The remaining cases were located in the right lower limb (23.3%) and upper limbs (1.04%). Among the DVT 287 cases, 69 (24.04%) developed pulmonary thromboembolism, and 18 (6.27%) experienced cerebral thrombosis.

Table 3 and Table 4 summarize the risk factors associated with pregnant/postpartum women concerning the occurrence of pulmonary embolism or cerebral thrombosis, for whom urgent transfer to a specialized internal medicine medical unit and neurology medical unit was requested. 

In all cases, it is apparent that surgical intervention via cesarean section is associated with a higher risk compared to vaginal delivery. Distinct risk factors were identified, such as obesity, diabetes, hypertension, preeclampsia, systemic lupus erythematosus, hereditary thrombophilia, and varicose veins, each contributing to varying extents (Table 5).

Overall, 0.31% of the 91,996 patients suffered from DVT. Furthermore, 24.04% of the 287 patients with DVT experienced cerebral thrombosis, while 6.27% developed a pulmonary embolism. 

Analyzing the risk factors in our DVT cohort, 201 out of 287 patients had associated risk factors, while 86 did not present with any. Among the 69 patients diagnosed with pulmonary embolism, 47 of them had associated risk factors; however, 22 patients with the aforementioned pathology did not exhibit any. Furthermore, among the 18 patients diagnosed with cerebral venous thrombosis, 12 had associated risk factors whilst 6 did not present with any.

(a) Univariate analysis of the risk for deep vein thrombosis (DVT)

The statistical analysis of risk factors for DVT revealed high significance for obesity (OR 3.676; CI 2.484–5.439), gestational diabetes (OR 3.394; CI 2.101–5.483), hypertension (OR 2.325; CI 1.591–3.397), preeclampsia (OR 4.753; CI 2.342–9.645), thrombophilia (OR 12.138; CI 8.973–16.417), and varicose veins (OR 9.678; CI 7.321–12.793) (Table 6).

(b) Univariate analysis of the risk for pulmonary thromboembolism

Our data analysis for pulmonary thromboembolism revealed high significance for obesity (OR 7.867; CI 4.297–14.401), hypertension (OR 2.605; CI 1.246–5.446), preeclampsia (OR 7.483; CI 2.346–23.872), thrombophilia (OR 11.035; CI 5.910–20.602), and varicose veins (OR 6.837; CI 3.665–12.757) (Table 7).

(c) Univariate analysis of the risk for cerebral thromboembolism (CTE)

However, for cerebral thromboembolism (CTE), the risk factors identified were obesity (OR 6.755; CI 1.954–23.347), hypertension (OR 1.167; CI 0.155–8.770), preeclampsia (OR 9.655; CI 1.283–72.672), and thrombophilia (OR 33.275; CI 12.884–85.939) (Table 8).

(d) Multivariate analysis of the risk factors

Based on our findings, we tried to provide a predictive model for the risk of DVT in pregnant and recently postpartum women using logistic regression, as shown in Table 9.

Combined risk factors analysis revealed that women with both obesity and hypertension had a significantly higher risk for DVT, 5.1% (95% CI: 3.9–6.5). When both hereditary thrombophilia and obesity risk factors are present, the risk of DVT becomes extremely high, 200 times higher compared to those without the abovementioned conditions. When analyzing the effect of hypertension associated with varicose veins, we observed that this combination of the two risk factors increases the risk for DVT by 80 times. A 60-times higher risk for DVT was noted in pregnant and recently postpartum women who presented with both preeclampsia and systemic lupus erythematosus. Women with both diabetes and hypertension are 13 times more likely to develop DVT. Further, when analyzing the birth type, obese women who gave birth via cesarean section are 33 times more likely to develop DVT.

In our study focusing on deep vein thrombosis (DVT) among pregnant patients, we observed that there were no cases of imminent miscarriage, amniotic fluid embolism, or fetal deaths. None of the multiple pregnancy patients suffered thrombotic events. However, out of the total patients, 20 (6.9%) experienced intrauterine growth restriction (IUGR). Among these, it is worth mentioning that two were twin pregnancies, three patients had varicose veins in the lower limbs as an associated risk factor, and two had thrombophilia (one of whom developed pulmonary thromboembolism). One of the included women had associated hypertension, and another one ended up developing preeclampsia. Nine (45%) out of the twenty women with IUGR fetuses presented with DVT and associated risk factors, while eleven of them had no associated risk factors.

One single case (0.34%) of placental abruption was documented in a patient at 21 weeks gestation, resulting in the loss of the pregnancy and the development of pulmonary thromboembolism; she had significant hepatic and cardiovascular pathologies as risk factors.

Three cases (1.04%) of miscarriage were detected at gestational ages of 7, 14, and 18 weeks, out of which two patients presented with pulmonary thromboembolism and only one presented with cerebral venous thrombosis. The woman who experienced a 7-week miscarriage developed viral encephalitis complicated by cerebral thrombosis. Meanwhile, the one with an 18-week miscarriage developed consumptive coagulopathy with thrombocytopenia and DVT, subsequently developing pulmonary thromboembolism and passing away.

## 4. Discussion

This retrospective study, spanning 15 years (2007–2021) and involving three major clinics in Iaşi County, provides a comprehensive analysis of the risk factors associated with DVT in pregnant and postpartum patients. This research aims to categorize patients with DVT within specific groups, compare results with the existing literature, and identify the most prevalent pathologies involved in order to improve prevention. This study employed a comprehensive retrospective analysis covering 287 pregnant/postpartum patients diagnosed with DVT. The prevalence of thrombotic events depicted in our study was higher, 0.31%, than the results of Varrias et al. and Park et al., where this complication rate ranges from 0.025 to 0.1% of pregnancies [24,25].

The univariate analysis of the abovementioned risk factors relating to DVT revealed that obesity, diabetes, hypertension, preeclampsia, hereditary thrombophilia, and varicose veins show significant associations with an increased risk of thrombosis. Obesity presents the highest risk, followed by thrombophilia and preeclampsia. Obesity, a globally prevalent risk factor with an ascendant slope in terms of prevalence, has a documented incidence of 13%, particularly among women, and has been implicated in the pathogenesis of DVT and subsequent complications. Notably, the incidence rate of DVT for pregnant women with a BMI > 30 kg/m^2^ is reported to be 45.7 per 10,000, in contrast to 22 in pregnant women with a BMI of 20–22 kg/m^2^ [14,16]. A study conducted by Mahmoud A. et al. in 2023 supports this finding, indicating that 45.7 pregnant women with a BMI > 30 were associated with DVT in 10,000 cases, equivalent to 0.45% [21]. 

Systemic lupus erythematosus does not demonstrate a statistically significant association with DVT risk when compared to other results available in the literature, which is likely due to a combination of factors [14,20,21]. These factors include the administration of efficient and closely monitored anticoagulant treatments during pregnancy, with monthly evaluations for each pregnant patient, as well as the potential underreporting of the condition. Further extensive studies are required to confirm or refute this hypothesis, and our results need to be carefully analyzed and interpreted.

The univariate analysis of the abovementioned risk factors relating to pulmonary thromboembolism revealed that obesity, hypertension, preeclampsia, hereditary thrombophilia, and varicose veins show significant associations with an increased risk of such thrombosis, while systemic lupus erythematosus does not indicate such an association. Gestational diabetes, which develops in 2–10% of pregnant women, increases the risk of thrombosis in deep venous territories by 28% compared to pregnant patients without diabetes [13,26]. Our study revealed its impact as a risk factor for TVP in univariate analysis only, exhibiting no impact at all concerning the risk of pulmonary thromboembolism or CTE. We believe that this interesting finding is due to the fact that gestational diabetes is transitorily manifested during pregnancy, lacking the vascular impairment of preexisting type 1 or 2 diabetes. Our results are similar to the ones highlighted by Won et al. and Park et al. but in total disagreement with most of the results available in the literature, such as those obtained by Gorar et al. and Deischinger et al. [13,25,27,28]. Some possible explanations reside in the non-autoimmune status of this particular type of diabetes compared with other types. This particular finding needs further analysis due to the fact that it is highly subject to statistical analysis errors relating to the low number of included cases, as well as the difficulty of establishing such a diagnosis during pregnancy, leading to the underestimated incidence of this major DVT complication [13,25,27,28].

The same aspects mentioned in the above paragraph become visible when analyzing the relationship between the risk factors and CTE. Obesity, hypertension, preeclampsia, and hereditary thrombophilia are associated with an increased risk of CTE, while systemic lupus erythematosus, varicose veins, and diabetes have no such association.

In the multivariate analysis, obesity is the only significant factor influencing the risk of DVT, pulmonary embolism, and CTE. Another noteworthy aspect is represented by the fact that hereditary thrombophilia is the main factor influencing the risk for pulmonary thromboembolism and CTE. Gestational diabetes and systemic lupus erythematosus show conflicting associations with DVT, pulmonary thromboembolism, and CTE, suggesting a more complex relationship between them; the underestimation of the incidence of the thrombotic disease or two abovementioned associated pathologies, or a lack of sufficient data to obtain a more reliable result. This requires further investigation, and our results regarding these two specific pathologies and thrombotic events need to be interpreted with caution.

The literature posits that 38% of diagnoses of deep vein thrombosis and related complications occur after delivery, with 63% specifying cesarean section and 37% after vaginal delivery [20,21,29]. Our study aligns closely with these percentages: 62.74% and 37.25% (Table 2).

Several factors may contribute to the observed elevated incidence of deep vein thrombosis (DVT) in our study, including the association of risk factors in 70.3% of patients with thrombosis. Notably, our risk factors agree with the literature, encompassing obesity, hypertension, diabetes, hereditary thrombophilia, and varicose veins in the lower limbs [20,21,29,30,31]. 

We detected the presence of multiparity or multiple pregnancies as a significant risk factor, which is in agreement with other literature studies, with increased thrombosis rates indicated in such cases. The trend observed in our study, marked by an increasing number of C-section births, may contribute to an elevated risk of venous thrombosis, suggesting the need for appropriate thromboprophylaxis based on risk categories [20,21,29,30,31,32]. The results of our study indirectly underline the importance of thorough thromboprophylaxis, especially in the context of surgical interventions. Because the preventative management of women following cesarean delivery in our hospitals assumes only three days of LMWH (low-molecular-weight heparin) in those without obvious risk factors, we feel compelled to emphasize these aspects.

Regarding the mean age of patients with venous thrombosis, the results of our study indicate an average age of 31.3 years. This mean age is quite lower than that reported in the literature (35 years), underlining our need to be more accurate in the triage of patients with risk factors that require close follow-up and preventive measures. In order to assess the statistical significance between patient age and the diagnosis of deep vein thrombosis, statistical tests were conducted, yielding the following outcome: according to the independent samples *t*-test, we found statistical significance underlined by a *p* value of less than 0.001 [2,3].

Concerning evaluating the impact of these analyzed risk factors, when comparing our results with those in the literature, besides the lack of association between systemic lupus erythematosus and thrombotic events in pregnancy and recent postpartum and the fact that gestational diabetes only increases the risk of DVT and not pulmonary and cerebral thrombosis, our results align. From the three major thrombotic risk factors described in the literature, our research identified all of them as having major impacts on DVT risk. The two most important risk factors were obesity and hereditary thrombophilia [33,34]. Distinct from the results available in the literature, which consider it to be a weak risk factor, we detected a higher incidence of DVT in women with varicose veins, with a 2.639 coefficient and 14 OR [35]. One other major risk factor for DV described in the literature is cesarean section birth [36]. Our analysis found it to be highly associated with DVT but with less power as a risk factor. In accordance with the results in the literature, not only hypertension in pregnancy but also preeclampsia seems to have a greater impact on DVT risk, with an OR of 9 and a 2.197 coefficient [37].

We detected an approximately 10% incidence of post-thrombotic syndrome. Post-thrombotic syndrome, characterized by venous stress resulting from the long-term effects of prior deep vein thrombosis, encompasses diverse manifestations, such as pain, discomfort, edema, and venous ulcers, adversely impacting the quality of life. In pregnancy, post-thrombotic syndrome occurs in 20–40% of women with DVT, with compression stockings effectively lowering its frequency by 50% [16]. In our investigation, the incidence noted was much lower compared to the numbers reported in the existing literature. We may then hypothesize that this difference results from the limited addressability and accessibility to healthcare services of patients within our study cohort.

These findings may be particularly important in clinical practice in terms of identifying and accurately quantifying the risks associated with DVT. The positive correlations between the identified risk factors and thrombotic pathology emphasize the importance of carefully monitoring the risk groups identified in our research. The practical implications of these findings could guide prevention and management efforts relating to DVT among patients with specific medical conditions, thereby contributing to improving the quality of medical care.

The results obtained raise awareness about the faulty management of some cases, especially concerning women with less obvious risk factors. All pregnant and postpartum patients with venous insufficiency or varicose veins, gestational diabetes, obesity, thrombophilia, and hypertension need prolonged DVT prophylaxis. The heightened prevalence of deep vein thrombosis associated with specific risk factors underscores the need for meticulous attention to thromboprophylaxis strategies to effectively mitigate potentially life-threatening complications, highlighting the crucial requirement for vigilant monitoring and the proactive management of this risk. The strengths of our study reside in the fact that it is the second one carried out in our country dealing with this subject, but with a larger number of thrombotic cases analyzed. Another aspect that raises its value is represented by the fact that it offers a global view of many risk factors that, more or less, impact the risk of DVT in pregnancy and recent postpartum, with the possibility of underlining our region’s particularities.

Our study has some limitations. Within our urban locale, an array of additional medical facilities beyond the three referenced in this work, as well as private clinics staffed by specialists adept in the treatment of deep vein thrombosis (DVT), exist. Hospitals typically admit only those afflicted with exacerbated DVT cases and, invariably, individuals afflicted with pulmonary or cerebral thrombosis. We posit that this underlying factor serves as the primary explanation for the observed incongruity, that is, the high incidence of these two compared to deep vein thrombosis. This discrepancy may be attributed to the fact that the symptomatology is non-specific, especially for pregnant patients, who may consider these clinical manifestations to be overlapping with pregnancy. Unfortunately, in a small percentage of patients, the first and only manifestation of DVT is pulmonary/cerebral embolism. Therefore, this can make DVT diagnosis difficult and delay treatment, with some patients developing pulmonary embolism. The potential influence of patient-related factors, such as a lack of awareness regarding emerging symptoms or non-adherence to prescribed treatment and thromboprophylaxis regimens, should also be considered. The limited number of cases with thrombotic disorders that we succeeded in identifying among such a broad general population represented another limitation of this study. This low number of cases with thrombotic events results in a high risk of statistically weak conclusions, especially for the subgroup analyses for pulmonary embolism and cerebral venous thrombosis. There is also the following to consider: the lack of formal sample size calculation with the risk of obtaining underpowered conclusions and the lack of randomization, introducing potential sampling bias.

## 5. Conclusions

In conclusion, our study clearly underlines the impact of various pathologies on the development of deep vein thrombosis during pregnancy in our region.

In our study cohort, 201 out of 287 patients with DVT had associated risk factors, while 86 presented with none. Of the 201 patients with risk factors, 47 presented pulmonary embolism, and 12 patients had cerebral venous thrombosis. Post-thrombotic syndrome had an incidence of approximately 10%.

Obesity was the only significant factor influencing the risk of DVT, pulmonary embolism, and CTE, and hereditary thrombophilia was the main factor influencing the risk of pulmonary thromboembolism and CTE. Systemic lupus erythematosus and gestational diabetes revealed conflicting results that need further investigation.

## Figures and Tables

**Table 1:** General characteristics of the extended study group.

General Characteristics	N (%)
Age (mean)	28.2 ± 5.9 years
Urban area	45,305 (49.24%)
Rural area	46,691 (50.75%)
Multiple pregnancies	1469 (1.59%)
Twin pregnancies	1429 (1.55%)
Triplet pregnancies	38 (0.04%)
Singleton pregnancy patients	32,456 (35.27%)
Recent postpartum patients (singleton pregnancies)	59,540 (64.73%)
Vaginal birth	29,200 (49.09%)
C-section birth	30,340 (50.87%)

**Table 2:** General characteristics of the deep vein thrombosis group.

General Characteristics Deep Vein Thrombosis Group	N (%)
Age (mean)	31.3 ± 6.78 years
Rural area	155 (54%)
Urban area	132 (45.99%)
Multiparous (more than 4 births)	48 (16.72%)
Multiple pregnancy	11 (3.83%)
Singleton pregnancy patients	185 (64.45%)
Recent postpartum patients (singleton pregnancies)	102 (35.54%)
Vaginal births	38 (37.25%)
C-section births	64 (62.74%)
Mortality	2 (0.69%)
Upper limb	3 (1.04%)
Right lower limb	67 (23.3%)
Left lower limb	130 (45.2%)
Pulmonary embolism	69 (20.4%)
Cerebral thrombosis	18 (6.27%)

**Table 3:** Risk factors associated with pregnant/postpartum women with pulmonary embolism.

Risk Factors Associated with Pulmonary Embolism	N (%)
Pregnant women	28 (40.57%)
Gestational age (mean) week	19.21
Early postpartum women (singleton pregnancies)	41 (59.42%)
Postpartum day (mean)	16.6
Vaginal birth	13 (31.7%)
C-section birth	28 (68.29%)
Obesity	13 (18.8%)
Gestational diabetes	1 (1.44%)
Hypertension	8 (11.59%)
Preeclampsia	3 (4.34%)
Systemic lupus erythematosus	1 (1.44%)
Hereditary thrombophilia	9 (13.04%)
Inferior limb varicose veins associated with venous insufficiency	12 (17.39%)

**Table 4:** Risk factors associated with pregnant/postpartum women with cerebral thrombosis.

Risk Factors Associated with Cerebral Thrombosis	N (%)
Pregnant women	4
Gestational age week (mean)	20
Early postpartum women (singleton pregnancies)	14
Postpartum day (mean)	8.5
Vaginal birth	4 (22.22%)
C-section birth	10 (71.42%)
Obesity	3 (16.6%)
Gestational diabetes	0
Hypertension	1 (5.55%)
Preeclampsia	1 (5.55%)
Systemic lupus erythematosus	0
Hereditary thrombophilia	7 (38.88%)
Inferior limb varicose veins associated with venous insufficiency	0

**Table 5:** Risk factors associated with deep vein thrombosis, pulmonary embolism, and cerebral thrombosis.

Risk Factors	Risk Factors Encountered in the Entire Lot of Patients Included (91,996 Patients)	Deep Vein Thrombosis Patients That Have Risk Factors	Pulmonary Embolism Patients That Have Risk Factors	Cerebral Thrombosis Patients That Have Risk Factors
	N (%)	N (%)	N (%)	N (%)
Obesity	2648 (2.87%)	28 (9.75%)	13 (18.8%)	3 (16.6%)
Gestational diabetes	1791 (1.94%)	18 (6.27%)	1 (1.44%)	0
Hypertension	4415 (4.79%)	30 (10.45%)	8 (11.59%)	1 (5.55%)
Preeclampsia	558 (0.60%)	8 (2.78%)	3 (4.34%)	1 (5.55%)
Systemic lupus erythematosus	84 (0.091%)	1 (0.34%)	1 (1.44%)	0
Hereditary thrombophilia	1730 (1.88%)	50 (17.42%)	9 (13.04%)	7 (38.88%)
Inferior limb varicose veins associated with venous insufficiency	2758 (2.99%)	65 (22.64%)	12 (17.39%)	0
Total	13,984 (15.2%)	201 (70.03%)	47 (68.11%)	12 (66.66%)

**Table 6:** Univariate analysis of the risk factors associated with deep vein thrombosis.

Risk Factor	Odds Ratio (OR)	Significance (*p*-Value)	Confidence Interval (CI)
Obesity	3.676	<0.001	2.484–5.439
Gestational diabetes	3.394	<0.001	2.101–5.483
Hypertension	2.325	<0.001	1.591–3.397
Preeclampsia	4.753	<0.001	2.342–9.645
Systemic lupus erythematosus	3.860	0.180	0.536–27.821
Hereditary thrombophilia	12.138	<0.001	8.973–16.417
Varicose veins	9.678	<0.001	7.321–12.793

**Table 7:** Univariate analysis of the risk factors associated with pulmonary thromboembolism.

Risk Factor	Odds Ratio (OR)	Significance (*p*-Value)	Confidence Interval (CI)
Obesity	7.867	<0.001	4.297–14.401
Gestational diabetes	0.741	No significant effect (0.766)	0.103–5.336
Hypertension	2.605	0.011	1.246–5.446
Preeclampsia	7.483	<0.001	2.346–23.872
Systemic lupus erythematosus	0.997	Lower risk	Not applicable
Hereditary thrombophilia	11.035	<0.001	5.910–20.602
Varicose veins	6.837	<0.001	3.665–12.757

**Table 8:** Univariate analysis of the risk factors associated with cerebral thrombosis.

Risk Factor	Odds Ratio (OR)	Significance (*p*-Value)	Confidence Interval (CI)
Obesity	0.003	<0.001	1.954–23.347
Gestational diabetes	0	Lower risk (0.989)	Not applicable
Hypertension	0.881	<0.001	0.155–8.770
Preeclampsia	9.655	<0.001	1.283–72.672
Systemic lupus erythematosus	0	Lower risk	Not applicable
Hereditary thrombophilia	33.275	<0.001	12.884–85.939
Varicose veins	0	No significant effect (0.987)	Not applicable

**Table 9:** Impact of risk factors on the likelihood of developing DVT.

Risk Factor	Coefficient	Odds Ratio	Interpretation (Times More Likely to Develop DVT)
Age	0.037	1.04	Minimal impact
Gestational age	0.015	1.02	Minimal impact
C-section	1.188	3.28	3.3
Obesity	2.301	10.00	10
Gestational diabetes	0.860	2.36	2.4
Hypertension	1.739	5.69	5.7
Preeclampsia	2.197	9.00	9
Systemic lupus erythematosus	1.891	6.62	6.6
Hereditary thrombophilia	3.007	20.22	20
Inferior limb varicose veins associated with venous insufficiency	2.639	14.00	14

## Data Availability

The data used to support the findings of this study are available upon request.
